# Contribution of cognitive function and fatigue to health-related quality of life in patients with *IDH*-mutant gliomas from diagnosis to long-term follow-up

**DOI:** 10.1093/nop/npag049

**Published:** 2026-05-24

**Authors:** Isabelle Rydén, Dongni Buvarp, Alice Neimantaite, Louise Carstam, Dima Harba, Lotta Weyhenmeyer, Andrew Lycett, Sigrid Malmqvist, Anneli Ozanne, Erik Elgeskog, Alba Corell, Tomás Gómez Vecchio, Anja Smits, Asgeir Store Jakola

**Affiliations:** Department of Clinical Neuroscience, Institute of Neuroscience and Physiology, Sahlgrenska Academy, University of Gothenburg, Gothenburg, Sweden; Department of Neurology, Sahlgrenska University Hospital, Gothenburg, Region Västra Götaland, Sweden; Department of Clinical Neuroscience, Institute of Neuroscience and Physiology, Sahlgrenska Academy, University of Gothenburg, Gothenburg, Sweden; Department of Neurology, Sahlgrenska University Hospital, Gothenburg, Region Västra Götaland, Sweden; Department of Clinical Neuroscience, Institute of Neuroscience and Physiology, Sahlgrenska Academy, University of Gothenburg, Gothenburg, Sweden; Department of Clinical Neuroscience, Institute of Neuroscience and Physiology, Sahlgrenska Academy, University of Gothenburg, Gothenburg, Sweden; Department of Neurosurgery, Sahlgrenska University Hospital, Gothenburg, Region Västra Götaland, Sweden; Department of Clinical Neuroscience, Institute of Neuroscience and Physiology, Sahlgrenska Academy, University of Gothenburg, Gothenburg, Sweden; Department of Clinical Neuroscience, Institute of Neuroscience and Physiology, Sahlgrenska Academy, University of Gothenburg, Gothenburg, Sweden; Department of Clinical Neuroscience, Institute of Neuroscience and Physiology, Sahlgrenska Academy, University of Gothenburg, Gothenburg, Sweden; Department of Clinical Neuroscience, Institute of Neuroscience and Physiology, Sahlgrenska Academy, University of Gothenburg, Gothenburg, Sweden; Institute of Health and Care Sciences, Sahlgrenska Academy, University of Gothenburg, Gothenburg, Sweden; Department of Neurology, Sahlgrenska University Hospital, Gothenburg, Region Västra Götaland, Sweden; Department of Neurosurgery, Sahlgrenska University Hospital, Gothenburg, Region Västra Götaland, Sweden; Department of Clinical Neuroscience, Institute of Neuroscience and Physiology, Sahlgrenska Academy, University of Gothenburg, Gothenburg, Sweden; Department of Clinical Neuroscience, Institute of Neuroscience and Physiology, Sahlgrenska Academy, University of Gothenburg, Gothenburg, Sweden; Institute of Health and Care Sciences, Sahlgrenska Academy, University of Gothenburg, Gothenburg, Sweden; Department of Clinical Neuroscience, Institute of Neuroscience and Physiology, Sahlgrenska Academy, University of Gothenburg, Gothenburg, Sweden; Department of Clinical Neuroscience, Institute of Neuroscience and Physiology, Sahlgrenska Academy, University of Gothenburg, Gothenburg, Sweden; Department of Neurosurgery, Sahlgrenska University Hospital, Gothenburg, Region Västra Götaland, Sweden

**Keywords:** Glioma, IDH mutation, Cognitive, HRQoL, Fatigue

## Abstract

**Background:**

Cognitive impairment, fatigue, and reduced health-related quality of life (HRQoL) are common in patients with *IDH*-mutant gliomas. However, it remains unclear which cognitive domains are most relevant for global HRQoL, how fatigue relates to these associations, and whether this differs across disease stages. This cross-sectional study examined associations between impairments in cognitive domains and global HRQoL across the disease trajectory.

**Methods:**

Objective neuropsychological test results from patients with *IDH*-mutant gliomas were evaluated preoperatively, at 1 year, and long-term (≥ 5 years). Results were classified into 6 cognitive domains. HRQoL was assessed using the EORTC QLQ-C30 global health score. Hierarchical regression models were applied at each time point, adjusting for age and sex. Sensitivity analyses examined the contribution of fatigue and anxiety/depression.

**Results:**

Preoperatively (*n* = 106 patients), impairments in cognitive domains significantly explained additional variance beyond demographic variables (Δ*R*² = 0.20, *p* < .001), with impairments in learning/memory associated with lower global HRQoL (*p* < .001). At 1 year (*n* = 106 patients), cognitive domains also explained additional variance (Δ*R*  ² = 0.16, *p* < .01), with language (*p* < .05) and executive impairments (*p* < .01) as significant predictors. In the long-term cohort (*n* = 82 patients), cognitive domains explained additional variance (Δ*R*² = 0.31, *p* < .001), with executive impairments showing the strongest association (*p* < .001). Fatigue was strongly associated with global HRQoL across time points (*p* < .001). Anxiety/depression showed weaker associations.

**Conclusions:**

Associations between cognitive functioning and global HRQoL differ across time points, with executive impairments showing the strongest associations. Fatigue represents an important co-occurring factor. These symptoms are potentially modifiable, indicating that rehabilitation may improve patients’ HRQoL.

Key Points

- Impairments in cognitive domains relate differently to global health-related quality of life (HRQoL) across disease stages in patients with *IDH*-mutant glioma.
- Learning and memory dominate at diagnosis and executive function at long-term.
- Fatigue is strongly associated with global HRQoL.

Importance of the Study

Cognitive impairment, fatigue, and reduced health-related quality of life (HRQoL) are common in patients with *IDH*-mutant gliomas. However, it remains unclear which particular cognitive domains are relevant for global HRQoL and whether this differs at different disease stages.

By examining objective domain-specific cognitive functioning at different time points, this study demonstrates that impairments in different cognitive domains are associated with global HRQoL at different stages of the disease. Learning and memory are most relevant at diagnosis, whereas executive dysfunction shows the strongest association with HRQoL over time. These findings highlight that executive function should be considered beyond more readily apparent domains such as memory and language, especially when evaluating long-term outcomes. In addition, fatigue shows robust associations with HRQoL. Fatigue and cognitive impairments represent potentially modifiable factors for rehabilitative interventions that could improve patients’ perceived HRQoL.

*IDH*-mutant gliomas are diffuse brain tumors that primarily affect younger adults, with an age at onset around 35-40 years.^1^ The incidence is approximately 0.5 - 0.8 per 100 000 per year, and this stage of life typically involves family responsibilities, careers, and other social and professional demands.^1^^,^^2^ Compared to *IDH*-wild-type gliomas, these patients have a favorable prognosis, with median surviving often exceeding 10 years, but still typically die at a younger age.

Both cognitive impairment and reduced health-related quality of life (HRQoL) are common and burdensome in patients with *IDH*-mutant gliomas.^3-8^ Their impact is especially ­relevant in patients with slow-growing tumors, who are often diagnosed at a stage of life associated with substantial cognitive, occupational, and social demands.

A systematic review in patients with low-grade glioma not only identified multiple factors associated with HRQoL, including fatigue, but also highlighted substantial heterogeneity and limited clarity regarding the relative importance of the identified factors.^9^ Long-term longitudinal data remain limited, but existing studies suggest that HRQoL may remain relatively stable over time, while symptoms such as fatigue and depression persist.^10^

Early multimodal treatment of *IDH*-mutant gliomas is common and typically involves both surgery and chemoradiotherapy. Prolonged survival increases patients’ exposure to cognitive impairments over extended periods.^11-13^ Although concordance between subjective cognitive complaints and objective test performance is generally low, both have been associated with HRQoL in patients with low-grade gliomas.^4^^,^^7^^,^^14^ Given the limited correspondence between perceived and measured cognitive impairments, objective neuropsychological assessment provides a more accurate estimate of patients’ actual cognitive functioning when evaluating cognition in relation to HRQoL. This is essential for targeting appropriate cognitive domains in rehabilitation.

Cognitive impairments are likely experienced differently during the disease trajectory. Early in the disease course, crisis reactions, a sense of gratitude for living and survival focus may make cognitive difficulties less apparent in daily life, while some cognitive impairments or their functional consequences might not yet have emerged. After treatment, as the focus transitions from survival to returning to work and resuming a normal life, cognitive impairments often become more prominent as demands increase and priorities shift. Over time, factors such as intensified treatment for tumor progression or long-term treatment-related adverse effects may then become increasingly relevant, further affecting functioning and subsequently HRQoL.^15-19^ In addition, response shift can be seen in HRQoL whereby the same patient weigh similar cognitive symptoms differently over time.^20^^,^^21^ Changes in perception can manifest as stable or changed HRQoL over time despite persistent symptoms or functional limitations and may reduce observed changes between time points as patients retrospectively reevaluate their prior health.^22^ In a study on glioma patients, no response shift was seen at the group level, but became clear in patients whose HRQoL changed.^23^

Patients are affected at different time points, across different cognitive domains, and with considerable variation in the severity of symptoms.^19^^,^^24^^,^^25^ This is an effect of multiple factors such as the specific neural networks affected, lesion momentum, and treatment-related effects.^15^^,^^18^^,^^19^^,^^25^^,^^26^ How impairments in specific cognitive domains relate to HRQoL remains unclear, and potential variation across time points are poorly understood. Identifying variables that are potentially modifiable or amenable to intervention, such as cognitive impairments, fatigue, and mental health issues may offer opportunities to improve or preserve HRQoL in patients with *IDH*-mutant gliomas.

The aim of this study was to identify how different aspects of cognitive functioning relate to patients’ perceived global HRQoL at different phases of the disease. Secondary analyses explored the potential contribution of fatigue and symptoms of anxiety/depression. We hypothesized that different objectively measured cognitive functions would relate to patients’ self-reported HRQoL but that the importance of specific functions could vary over time.

## Methods

### Participants

Patients were identified at multidisciplinary team conferences held at Sahlgrenska University Hospital, Sweden, between February 2017 and December 2025. Adult patients scheduled for surgical resection or biopsy, with a radiological presentation suggestive of a diffuse low-grade glioma (typically a hyperintense lesion without pronounced contrast enhancement on T2-FLAIR Magnetic Resonance Imaging sequences), were invited to participate. Participants were preoperatively assessed in person by a neuropsychologist. Self-report questionnaires were distributed to be completed prior to the visit. Any questions were addressed during the assessment and all patients provided written informed consent.

Molecular diagnostics followed current clinical standards. *IDH* mutations were first analyzed by immunohistochemistry, with next-generation sequencing in negative or inconclusive cases. The 1p/19q codeletion status was determined by either DNA methylation-based copy number analysis or by fluorescence in situ hybridization.^27^ Only patients with a confirmed *IDH*-mutant grade 2-4 astrocytoma or oligodendroglioma (WHO 2021 classification) were eligible.

**Figure 1.**
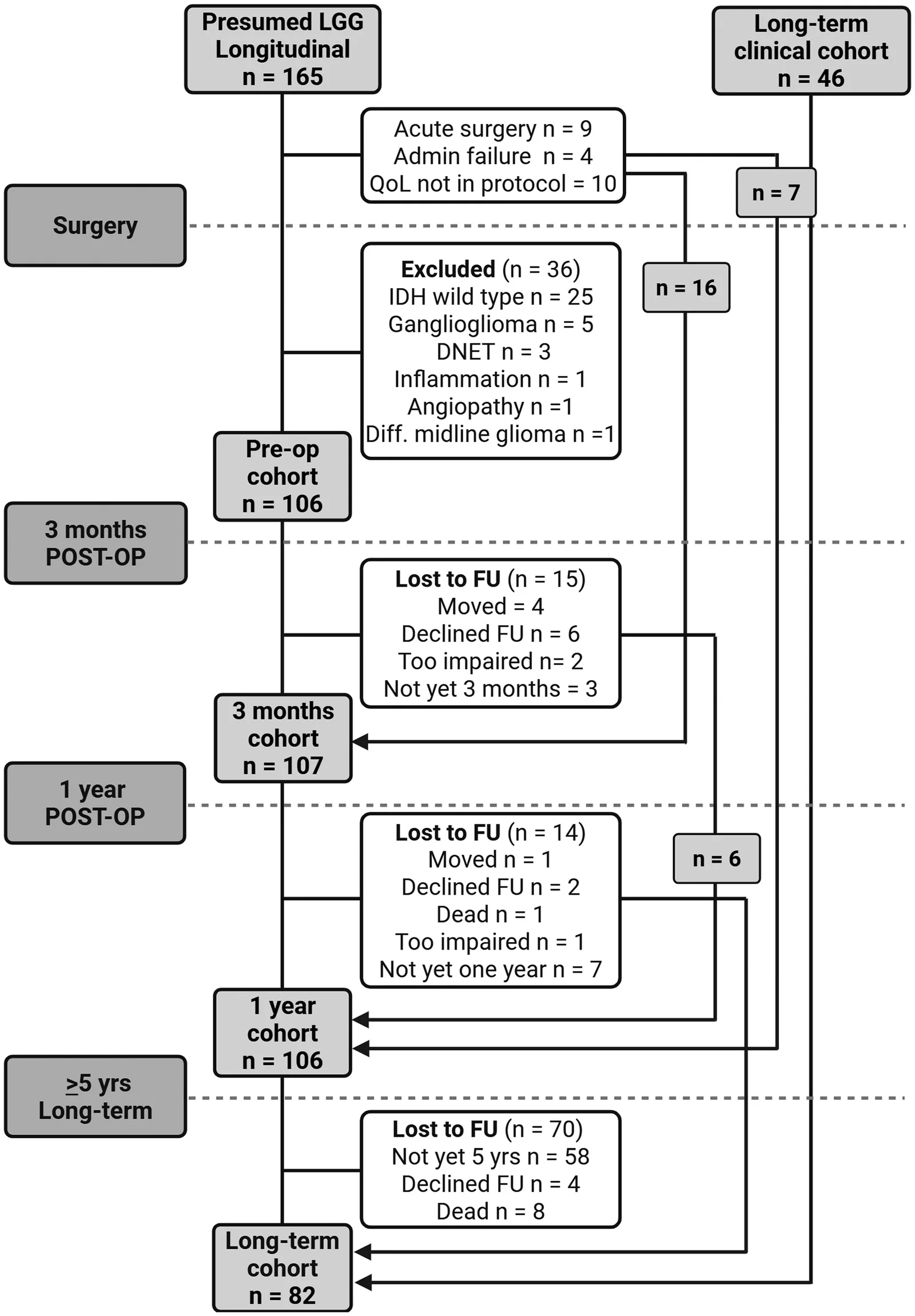
Flow chart of patient inclusion. Created in BioRender. Denes, A. (2026) https://BioRender.com/fuaxuxr. Abbreviations: FU = follow-up, LGG = low-grade glioma, QoL = quality of life.

Patients assessed preoperatively were followed and invited to standardized follow-up assessments at 3 months, 1 and 5 years postoperatively. In addition, patients who were not assessed preoperatively (usually because of acute procedures or logistical constraints) were invited to participate at the earliest feasible postoperative time point and were thereafter followed according to the same schedule. Patients who had not yet reached their scheduled follow-up time points and therefore only had preoperative data available were also included. Furthermore, patients with a confirmed *IDH*-mutant glioma diagnosed before 2017 were invited. These patients were typically identified via the neurology outpatient clinic and were included in the long-term cohort, resulting in a cohort of patients ranging from 5 to 27 years since diagnosis. This cross-sectional inclusion strategy was applied to minimize patient exclusion. As many patients were assessed at multiple time points, the cohorts largely overlapped. The study design results in 4 partly overlapping cohorts: preoperative, 3-month postoperative, 1-year postoperative and long-term (≥5 years). A flow chart of the inclusion process is presented in Figure 1.

Demographic, clinical, and treatment data were retrieved from electronic medical records. Tumor volumes were estimated from semi-automated segmentations of preoperative and early postoperative (<72 h postoperatively) T2-FLAIR images. Neurological symptoms were systematically documented, and focal deficits persisting beyond 3 months after surgery were considered permanent. Permanent deficits were categorized as major, if daily functioning was substantially impaired, including restrictions of work, driving, travel, and/or physical activity, with consequent inability to maintain a normal socio-professional life.^28^ These data are presented in Table 1.

**Table 1.** Background characteristics per patient group.

Variables	Preoperative (*n* = 106)	Three months (*n* = 107)	One year (*n* = 106)	Long-term (*n* = 82)
Age, M (SD)	43.6 (13.1)	43.2 (12.4)	43.5 (11.7)	47.6 (10.8)
Sex, male, *n* (%)	56 (52.8)	56 (52.3)	58 (54.7)	45 (54.9)
Education, M (SD), years	13.5 (3.1)	13.8 (2.9)	13.9 (2.9)	13.5 (2.9)
Epilepsy at disease onset, *n* (%)	73 (68.9)	67 (62.6)	70 (66.0)	58 (70.7)
AED use, *n* (%)	69 (65.1)	65 (60.7)	72 (67.9)	56 (68.3)
HADS, *n* (%)				
Anxiety ≥8	41 (38.7)	34 (31.8)	24 (22.6)	24 (30.0)
Depression ≥8	11 (10.4)	12 (11.2)	15 (14.2)	12 (15.0)
Main lobe, nr (%)				
Frontal	58 (54.7)	58 (54.2)	53 (50.0)	55 (67.1)
Temporal	21 (19.8)	22 (20.6)	25 (14.2)	9 (11.0)
Other	27 (25.5)	27 (25.2)	28 (26.4)	18 (22.0)
Hemisphere, left nr (%)	49 (46.2)	47 (43.9)	50 (47.2)	37 (45.1)
Preop. tumor volume, Mdn (Q1-Q3), ml	46.9 (63.3)	46.8 (71.8)	46.1 (71.3)	45.0 (81.1)
Resective surgery, nr (%)	99 (93.4)	104 (97.2)	101 (95.3)	82 (100.0)
Awake surgery, *n* (%)	29 (27.4)	29 (27.1)	28 (26.4)	13 (15.9)
RANO resect class, *n* (%)^a^				
1	3 (3.0)	2 (1.9)	3 (3.0)	2 (2.7)
2	48 (48.5)	55 (52.9)	52 (51.5)	36 (49.3)
3	38 (38.4)	36 (34.6)	36 (35.6)	25 (34.2)
4	10 (10.1)	11 (10.6)	10 (9.9)	10 (13.7)
WHO 2021 grade at disease onset, *n* (%)				
Astrocytoma 2	33 (31.1)	32 (29.9)	35 (33.0)	18 (22.0)
Astrocytoma 3	15 (14.2)	14 (13.1)	15 (14.2)	12 (14.6)
Astrocytoma 4	11 (10.4)	13 (12.1)	10 (9.4)	3 (3.7)
Oligodendroglioma 2	25 (23.6)	27 (25.2)	27 (25.5)	24 (29.3)
Oligodendroglioma 3	22 (20.8)	21 (19.6)	19 (17.9)	25 (30.5)
Postop. major permanent deficit, *n* (%)	-	5 (4.7)	3 (2.8)	2 (2.4)
Any cognitive impairment	50 (47.2)	-	50 (47.2)	43 (51.2)
Total fatigue score, Mdn (Q1-Q3)	55 (39-67)	51 (36-67)	51 (36-70)	58 (42-70)
Global HRQoL score, M (SD)	58 (22)	63 (21)	65 (23)	63 (23)
Radiotherapy before assessment, *n* (%)				
No	–	61 (57.0)	30 (28.3)	8 (9.8)
Photon	–	26 (24.3)	35 (33.0)	39 (47.6)
Proton	–	20 (18.7)	41 (38.7)	35 (42.7)
Radiotherapy dose, Gy (fractions), *n* (%)				
60.0 (2 × 30)	–	9 (19.6)	7 (9.2)	18 (24.3)
59.4 (1.8 × 33)	–	21 (45.7)	25 (32.9)	19 (25.7)
54.0 (1.8 × 30)	–	15 (32.6)	33 (43.4)	25 (33.8)
50.4 (1.8 × 28)	–	0 (0.0)	9 (11.8)	12 (16.2)
40.05 (2.67 × 15)	–	1 (2.2)	2 (2.6)	0 (0.0)
Chemotherapy before assessment, *n* (%)				
No	–	75 (70.1)	23 (21.7)	10 (12.2)
Temozolomide	–	28 (26.2)	56 (52.8)	46 (56.1)
PCV	–	4 (3.7)	26 (24.5)	25 (30.5)
Lomustine	–	0 (0.0)	1 (0.9)	1 (1.2)
Stable disease at assessment, *n* (%)	–	104 (100)	101 (98.1)	65 (82.3)
Second-line treatment for recurrence/progression, *n* (%)	–	0	0	25 (30.5)
Re-operation before assessment, *n* (%)	–	1 (0.9)	2 (1.9)	27 (67.1)
Time since surgery, months (SD)	–	3.3 (1.5)	12.6 (1.9)	103.1 (48.9)

a At 3 months, *n* = 104; 1 year, *n* = 101; long-term, *n* = 73.

### Heatmaps of Tumor Locations

To visualize and compare spatial tumor distribution between patient cohorts at different time points, tumor location heatmaps were used. Tumor segmentations were spatially normalized to the Montreal Neurological Institute (MNI) space using previously described registration procedure.^29^^,^^30^ In case of errors in registration, manual adjustments were made in registration and/or corrected by landmark registration in 3D Slicer.^31^ Group-wise overlap maps were obtained by summing the registered segmentations, and colormaps scaled to the maximum overlap value were used. Applied software in the registration and creation of heatmap figures were Python programing language (v3.8.3) and 3D Slicer.^31^

### Instruments

#### Neuropsychological assessments

Neuropsychological assessments were conducted by a clinical neuropsychologist (primarily I.R.) and lasted approximately 2 h. Raw test scores were standardized to *z*-scores using published normative data. The neuropsychological tests were categorized into 6 cognitive domains: language (Boston Naming Test; Delis-Kaplan Executive Function System [D-KEFS] Verbal Fluency), learning/memory (Rey Auditory Verbal Learning Test; Brief Visuospatial Memory Test-Revised), executive functioning (Trail Making Test Part B; D-KEFS Color-Word Interference Test conditions 3-4), attention/working memory (Wechsler Adult Intelligence Scale-IV Digit Span), visuospatial functioning (Rey Complex Figure Test copying), and processing speed (Trail Making Test Part A; Wechsler Adult Intelligence Scale-IV Coding). Descriptions of the neuropsychological tests are provided in Table S1 and an overview of the tests comprising each domain are listed in Table S2. All tests were assessed at each time point, except at the 3 months follow-up when the Trail-Making Test, the Color Word Interference Test, and the Rey Complex Figure Test were not assessed. Domain-level cognitive impairment was further dichotomized and classified as present if either 2 tests within a domain were at least 1.5 SD below the normative mean or if one test was at least 2.0 SD below the normative mean, in accordance with recommendations from the International Cognition and Cancer Task Force (ICCTF).^32^ To further examine whether the presence of cognitive impairment per se was associated with global HRQoL, a dichotomous composite variable representing *any cognitive impairment* was created. Consistent with the domain-level classifications, an impairment was defined as at least one impaired cognitive domain (yes/no).

#### Patient-reported outcome measures

##### Health-related quality of life assessment

HRQoL was assessed using the European Organisation for Research and Treatment of Cancer (EORTC) Quality of Life Questionnaire (QLQ) Core questionnaire (C30), version 3. For the present study, the Global health status/QoL (global HRQoL) was used as outcome variable. The Global HRQoL score represents a validated and conceptually distinct measure of overall perceived health and quality of life, scored independently from symptom scales, thereby minimizing conceptual overlap with symptom-based measures.^33^^,^^34^ The global HRQoL was derived from the 2 items: “How would you rate your overall *health* during the past week?” and “How would you rate your overall *quality of life* during the past week?” with response options ranging from 1 (very poor) to 7 (excellent).

Given the established associations between HRQoL and patient-reported outcomes (PROs), analyses were conducted to examine their contribution as co-occurring factors.^35^^,^^36^ The PROs fatigue and depression/anxiety were selected based on their availability, clinical relevance and established associations with both HRQoL and cognitive function. The following patient-reported outcome measures (PROMs) were used for these analyses:

##### Mental fatigue assessments

Mental fatigue was assessed using the Multidimensional Fatigue Inventory (MFI-20), a validated self-report questionnaire comprising 20 items that evaluate 5 dimensions of fatigue: *general fatigue*, *physical fatigue*, *mental fatigue*, *reduced motivation*, and *reduced activity.* Each domain consists of 4 items rated on a 5-point Likert scale, yielding domain scores ranging from 4 to 20, with higher scores indicating greater fatigue severity. In addition to the domain scores, a total fatigue score (range 20-100) was calculated by summarizing all items.^37^ The total score was used in the regression analyses as a global indicator of overall fatigue burden. Missing data were handled by calculating domain scores when at least 50 % of items were completed; missing items were imputed using the individual means of the remaining domain items. In total, 8 scores (< 1%) were imputed.

##### Assessing depression and anxiety

Symptoms of depression and anxiety were measured with the Hospital Anxiety and Depression Scale (HADS).^38^ HADS consists of one subscale for anxiety, and one for depression, each based on 7 items rated on a 0-3 scale.^38^ For both subscales, a cutoff of ≥ 8 was applied to indicate clinically relevant symptoms.^39^^,^^40^ In regression analyses, a score above cutoff on either subscale (yes/no) was used to indicate elevated symptoms.

### Statistical Analyses

Background characteristics, tumor-related, treatment-related, and patient-reported variables were summarized using descriptive statistics. Statistical analyses were conducted in the Statistical Package for the Social Sciences (SPSS) version 28.0 (IBM Corp., Armonk, NY, USA). Two-tailed tests were applied, and results with *P*<.05 were regarded as statistically significant.

#### Analyses of associations between cognitive functioning and global HRQoL

To examine the association between objectively assessed cognition and HRQoL, separate hierarchical multiple linear regression analyses were conducted, one for each assessment time point. Global HRQoL was used as the dependent variable in all analyses. In Block 1, the demographic variables (age and sex) were entered. In Block 2, the 6 cognitive domains derived from the objective neuropsychological testing were added: language, learning and memory, executive function, attention, visuospatial ability, and processing speed. Regression models were evaluated for standard assumptions, and each variable required a minimum of 10 observations per predictor. Due to insufficient data across several cognitive domains (2 not assessed and 2 not meeting regression assumptions), cognitive analyses were not performed at the 3-month follow-up. Explained variance (*R*²) and changes in explained variance (Δ*R*²) were evaluated at each step.

Group comparisons of global HRQoL between patients with and without *any cognitive impairment* were conducted separately at each assessment time point. Due to the ordinal nature of the global HRQoL measure, non-parametric Mann-Whitney *U* tests were used when comparing patients in relation to *any cognitive impairment*.

#### Sensitivity analyses including patient-reported fatigue and anxiety/depression

Regression models, identical to those used for the cognitive domains, were applied for each time point, with PROs entered in place of the cognitive domains. In these analyses, demographic variables (Block 1) were retained; fatigue (MFI-20 total score) and depression/anxiety (HADS ≥ 8 on either subscale) were entered as a separate block (Block 2). The assumed close relationship between fatigue and symptoms of depression and anxiety was examined using Spearman correlations.

## Results

### Participants

**Figure 2.**
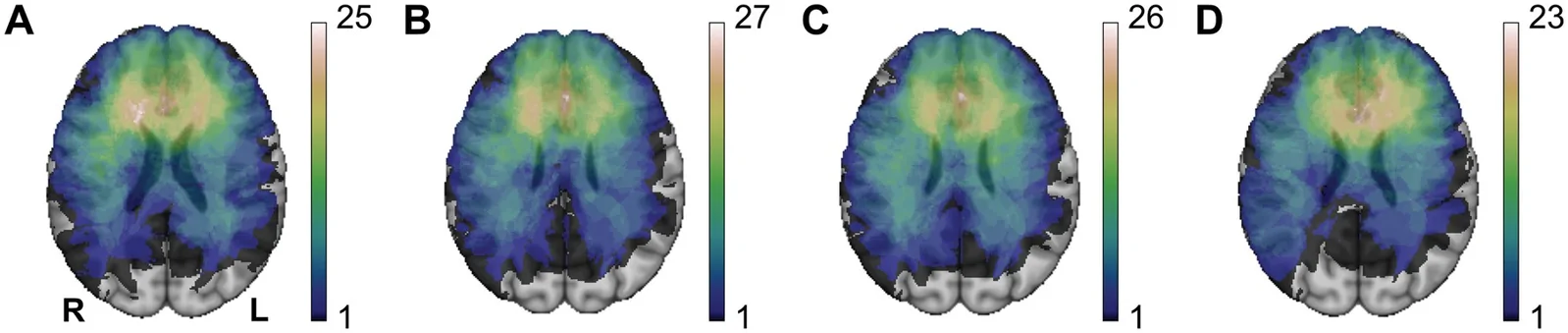
Heatmap of tumor location before first surgery in the cohorts: (A) preoperative (*n* = 106), (B) 3 months (*n* = 107), (C) 1-year (*n* = 106), and (D) long-term (*n* = 78*). * denotes preoperative MRI images were missing for 4 patients in the long-term cohort.

The inclusion process is presented in Figure 1. As illustrated, 106 patients were included preoperatively, 107 patients at 3 months, 106 patients at 1 year, and 82 patients in the long-term cohort. Background characteristics, tumor-related, treatment-related, and patient-reported variables are presented in Table 1. To summarize, over 90 % underwent surgical resection (as opposed to biopsy only). Sex distribution was balanced, with a slight predominance of men. Mean age at time of testing was similar across cohorts (approximately 43 years), although patients in the long-term follow-up cohort were older (M = 48 years). Clinically relevant symptoms of anxiety were more common preoperatively and depressive symptoms more prevalent at later time points. Patients assessed at later time points after initial surgery had more often received oncological treatment. Frontal tumor location was the most frequent location across all groups. In the long-term follow-up cohort, a larger proportion of patients had frontal tumors and oligodendrogliomas. Tumor locations are further illustrated in Figure 2 and in Figures S2-S5.

### Cognitive Functioning and Global HRQoL

The regression models examining the association between cognitive domains and global HRQoL are presented in Table 2. In the preoperative model, the speed domain was excluded due to too few impaired cases to allow reliable regression. Cognitive domains explained an additional 20% of the variance in HRQoL (after including age and sex in the first step), with impairments in the learning and memory domain significantly associated with lower HRQoL. At 1 year, the speed domain again did not meet inclusion criteria. Results indicated that increasing age was associated with lower global HRQoL, but demographic variables only accounted for 2% of the explained variance. The inclusion of cognitive domains explained an additional 16% of the variance in HRQoL. Language deficits and executive dysfunction were significantly associated with lower HRQoL. At long-term follow-up, age was a significant predictor, and demographics explained 11% of the variance in global HRQoL. The inclusion of cognitive domains explained an additional 31% of the variance in HRQoL, with executive dysfunction being the only significant predictor.

**Table 2.** Hierarchical regression models for cognitive domains in relation to global HRQoL preoperatively, 1 year postoperatively, and in the long-term cohort.

		Global HRQoL preoperative (*n *= 106)	Global HRQoL 1 year (*n* = 106)	Global HRQoL long-term (n = 82)
		β	β	β
Block 1	Sex	0.04	−0.01	0.21
	Age	0.01	0.20*	−0.29**
		Adj. *R*² = −0.02	Adj. *R*² = −0.02	Adj. *R*² = 0.11
Block 2	Domain Language	−0.04	−0.23*	0.08
	Domain Learning/memory	−0.42***	0.16	0.12
	Domain Executive	−0.06	−0.31**	−0.58***
	Domain Attention	−0.05	0.02	−0.13
	Domain Visuospatial	0.06	−0.11	−0.03
	Domain Speed	NA	NA	−0.09
		Adj. *R*² = 0.14	Adj. *R*² = 0.14	Adj. *R*² = 0.39
		ΔR² = 0.20***	ΔR² = 0.16**	ΔR² = 0.31***

* *p* < .05.

** *p* < .01.

*** *p* < .001. β, standardized beta coefficient; Adj. *R*  ², adjusted explained variance; NA, not available because assumptions were not met; Δ*R*  ², change in unadjusted *R*  ² between blocks (*F*-change test).

**Figure 3.**
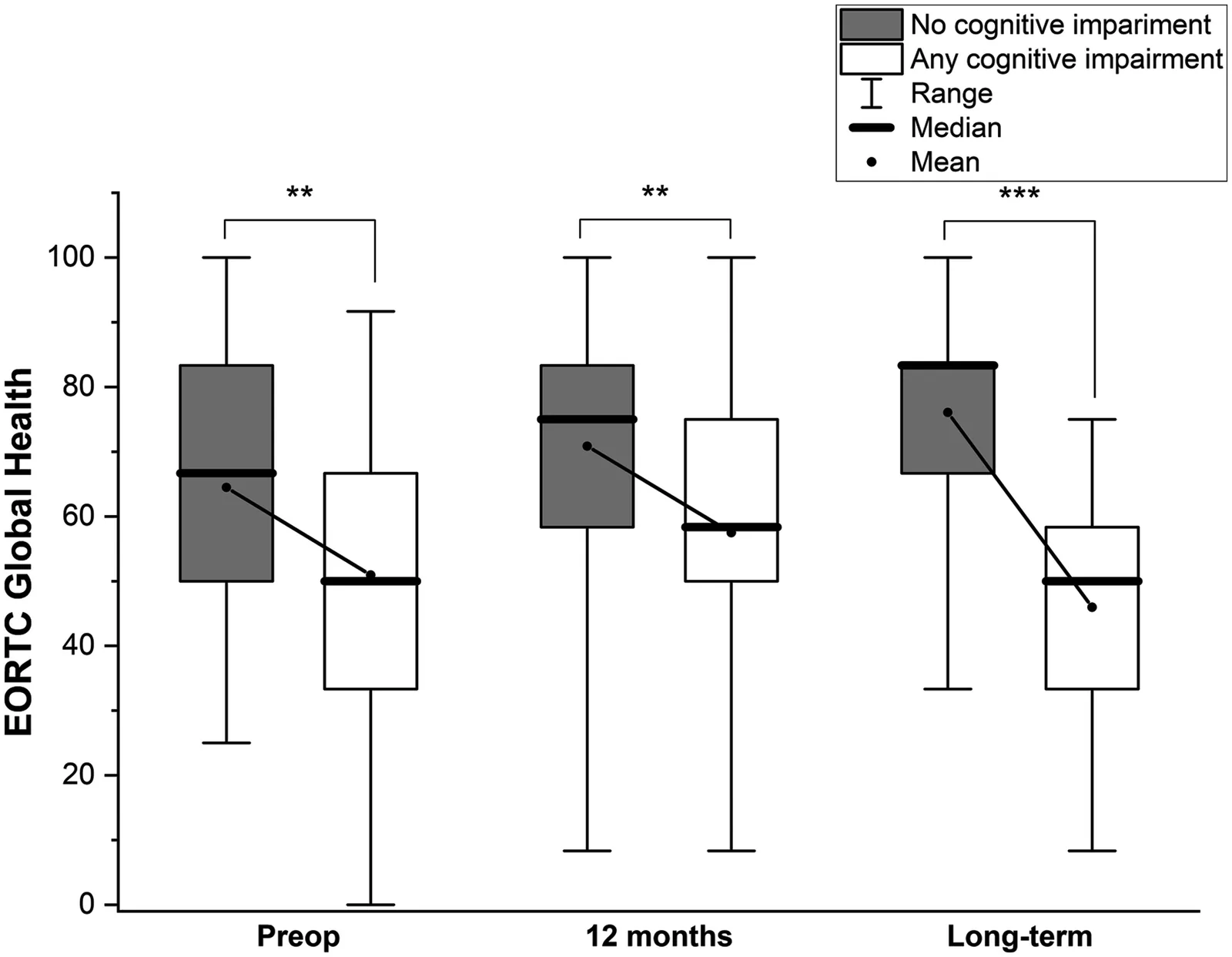
Global HRQoL stratified by presence of any present cognitive impairment at different assessment time points. Abbreviation: EORTC = European Organisation for Research and Treatment of Cancer.

Group comparisons based on the dichotomous classification of *any cognitive impairment* are presented in Figure 3. Patients with *any cognitive impairment* reported significantly lower global HRQoL preoperatively (Mann-Whitney *U* = 925, *Z* = −3.06, *p* = .002), at 1 year (*U* = 907, *Z* = −3.15, *p* = .002), and at long-term follow-up (*U* = 195, *Z* = −5.93, *p* < .001). Due to insufficient cognitive data at the 3-month assessment, this time point was excluded from the group comparisons.

#### Sensitivity analyses of patient-reported fatigue and anxiety/depression

The results of the hierarchical regression models are presented in Table 3. Fatigue emerged as the dominant PRO associated with global HRQoL across time points. Symptoms of depression or anxiety showed weaker and less consistent associations with HRQoL. However, the association between mental fatigue (total score) and symptoms of anxiety and depression as measured by HADS showed moderate to high correlations (ρ = 0.56-0.77). Given the prominence of fatigue in these analyses, we wanted to explore whether cognitive domains were differentially related to HRQoL in a more homogeneous subgroup of patients with higher fatigue burden. Patients were stratified by fatigue level using a median split of the total fatigue score. This method was used to ensure sufficiently large and balanced subgroups. Hierarchical regression analyses were then performed for the high-fatigue subgroup.

**Table 3.** Hierarchical regression models of self-reported fatigue and anxiety/depression in relation to global HRQoL preoperatively, 1 year postoperatively, and in the long-term group.

		Global HRQoL Preoperative (*n* = 106)	Global HRQoL 1 year (*n* = 106)	Global HRQoL Long-term (*n* = 82)
		β	β	β
Block 1	Sex	0.06	−0.03	0.27*
	Age	0.04	−0.21*	−0.26*
		Adj. *R*² = −0.02	Adj. *R*² = 0.03	Adj. *R*² = 0.12
Block 2	MFI total fatigue	−0.61***	−0.68***	−0.74***
	HADS depression/anxiety	−0.21*	−0.18*	−0.09
		Adj. *R*² = 0.53	Adj. *R*² = 0.62	Adj. *R*² = 0.61
		ΔR² = 0.54***	ΔR² = 0.59***	ΔR² = 0.49***

* *p* < .05.

** *p* < .01.

*** *p* < .001. β, standardized beta coefficient; Adj. *R*  ², adjusted explained variance; NA, not available as assumptions were not met; Δ*R* ² = change in unadjusted *R*  ² between blocks (*F*-change test).

Results from the regression analyses of patients in the high-fatigue group are shown in Table S3. Preoperatively, cognitive domains explained a substantial proportion of variance in HRQoL, with learning/memory remaining significant, and language emerging as an additional predictor in the high-fatigue group. At 1 year, executive function was the only significant cognitive domain. At long-term follow-up, executive dysfunction remained the sole significant cognitive predictor. Thus, stratification by fatigue did not substantially alter the overall pattern of associations, although language was significant at baseline in the high-fatigue group but not in the full cohort.

##### Associations of fatigue domains and HRQoL

Given the strong associations between HRQoL and fatigue, we further examined whether any specific fatigue dimension was more strongly related to global HRQoL. This was also motivated by the hypothesis that certain fatigue components, particularly mental fatigue, might demonstrate stronger associations with HRQoL than other dimensions, such as physical fatigue.^41^ Additional regression analyses were conducted in which the MFI-20 total score was replaced by its 5 subscales in Block 2. Although the fatigue dimensions were moderately to strongly intercorrelated (*r* = 0.49-0.74), collinearity diagnostics indicated acceptable levels, allowing simultaneous inclusion of all fatigue domains.

Results are shown in Table S4. Preoperatively, *physical fatigue* and *reduced motivation* were significantly associated with global HRQoL. At 3 months, *general fatigue*, *physical fatigue*, *mental fatigue*, and *reduced motivation* were all significant. At 1 year, *general fatigue*, *physical fatigue*, and *reduced motivation* were significant, and in the long-term cohort only *general fatigue* was significant.

##### Fatigue in relation to cognition

To investigate the relation between fatigue and cognitive functions, Spearman correlations between MFI total score, cognitive domains, and *any cognitive impairment* were performed. Results are presented in Figure S1A-C. Preoperatively, the total fatigue score was significantly correlated with impairments in learning and memory (ρ  =  0.29, *p* = .003) and executive functioning (ρ  =  0.20, *p* = .046). At 1 year, total fatigue was significantly correlated with impairments in language (ρ  =  0.26, *p* = .007), executive function (ρ  =  0.36, *p* < .001), and processing speed (ρ  =  0.22, *p* = .029) as well as *any cognitive impairment* in cognitive domains (ρ  =  0.28, *p* = .005). In the long-term cohort, total fatigue was significantly correlated with deficits across all cognitive domains (ρ  =  0.30-0.52, *p* = .007 − <.001) as well as the presence of cognitive impairment in any domain (ρ  =  0.53, *p* < .001).

## Discussion

In this study, we examined associations between cognitive impairment and global HRQoL across several stages of the disease. *Any cognitive impairment* was associated with lower global HRQoL at all time points, it was most pronounced in the long-term cohort, and the relevance of specific cognitive domains varied. Similar associations have also been reported in a cross-sectional study of long-term survivors with low-grade glioma, demonstrating that objective cognitive deficits are relevant for HRQoL several years after diagnosis.^5^ However, which specific cognitive domains contribute most to global HRQoL has not been clarified.

We observed that learning and memory were most strongly associated with global HRQoL at diagnosis, and that language was associated with global HRQoL at 1 year. In line with this, a qualitative study showed that patients specifically considered memory and communication central to their quality of life.^42^ Notably, that study included not only patients assessed early after treatment but also patients several years after diagnosis. Both memory and language have previously been found to be affected in patients with *IDH*-mutant gliomas, but these studies have not examined their association with HRQoL.^18^^,^^19^ A plausible interpretation of our result is that early in the disease course and around the time of surgery, global HRQoL may be more affected by easily identifiable difficulties, such as memory or language problems. Suggesting that early assessment and support targeting these cognitive domains may be particularly important at this stage. During this early phase, many patients are on sick leave, receive substantial support from others, and may not yet be exposed to the cognitive demands of work or independent daily functioning.^43^ Memory and language difficulties may nevertheless be experienced as distressing. Also, worry and anxiety may be more prominent at this stage and overshadow other symptoms.

In addition to language, executive impairments showed an even stronger association with global HRQoL at 1 year, and in the long-term cohort, executive functioning was the only significant predictor and showed a strong association. It has previously been shown that executive functioning is commonly affected at various stages of the disease ­trajectory.^15^^,^^17^^,^^19^^,^^44^^,^^45^ These difficulties are characterized, for example, by impairments in cognitive flexibility, set shifting, and inhibitory control. While they may not be readily recognized as a distinct impairment, they can nonetheless have substantial functional consequences, particularly as patients resume everyday activities.^15^ One year after initial surgery, more than half of patients have typically returned to work and may face increased cognitive demands, making executive problems more apparent and more likely to affect HRQoL.^43^^,^^45^ This may partly explain the stronger association between executive impairment and HRQoL later in the disease trajectory, but suggests a need for assessment of executive functioning in follow-up, particularly when patients resume work and independent daily activities.

*IDH*-mutant gliomas are typically present in the frontal and temporal lobes, which was also seen in our cohort. Our long-term cohort comprised a higher proportion of patients with oligodendroglioma, reflecting their more favorable prognosis. These gliomas show preferential involvement of frontal brain regions, a pattern we also observed in the detailed heatmaps of our long-term patients. Frontal lobe involvement is well known to be associated with executive dysfunction.^46^ Long-term treatment effects, cumulative treatment burden, and/or tumor progression affecting these regions may therefore contribute to increasing executive difficulties over time. Recently, a large cross-sectional multicenter study of long-term survivors with oligodendroglioma reported comparable global HRQoL levels (M = 67, SD = 23) to our long-term cohort (M = 63, SD = 23). The study also reported prevalent objective cognitive impairment, including deficits in memory, language, and executive functioning, but did not examine direct associations between cognitive impairment and global HRQoL.^17^ In the long-term cohort, age was also a significant predictor. This may reflect the longer time since initial treatment in this cohort, with increasing exposure to cumulative disease- and treatment-related sequelae.

### The Influence of Fatigue

In addition to cognitive impairments, fatigue was strongly associated with global HRQoL and was therefore examined further. Fatigue has also previously been associated with both cognitive functioning and symptoms of anxiety and depression.^5^^,^^47^ Recent reviews have identified fatigue and cognitive dysfunction as among the strongest and most clinically relevant determinants of HRQoL in patients with glioma, underscoring the importance of addressing these symptoms in both research and clinical care.^36^

Overall, associations between fatigue and cognition broadened and strengthened over time, from weak at earlier stages to stronger at later time points. It is possible that this widening pattern of associations over time is partly driven by the larger proportion of patients with both fatigue and cognitive impairments at that time point, rather than reflecting a qualitative change in its relationship with cognition. In an attempt to make the cohort more homogeneous with respect to fatigue, we ran a series of sensitivity analyses that largely confirmed the associations.

Our analyses of fatigue domains suggested that global HRQoL was primarily related to a more generalized experience of fatigue, rather than a specific fatigue component consistently driving this association. General fatigue, reflecting an overall sense of tiredness and lack of feeling rested, appeared most robustly related to HRQoL.

It has previously been shown that mental fatigue in low-grade glioma is closely associated with objective impairments in executive functioning.^47^ It was unexpected that mental fatigue was not more strongly or persistently associated with global HRQoL in our study, given that it is a common symptom and conceptually closely linked to cognitive functioning. A previous study in patients with *IDH*-mutant grade 2 glioma demonstrated that mental and physical fatigue differ not only in prevalence but also in their underlying determinants, with mental fatigue being more strongly associated with symptoms of anxiety and depression and impairments in higher-order cognitive processes.^41^ Overall, our results indicate that fatigue may be an important co-occurring factor in the relationship between cognition and self-perceived global HRQoL.

Identifying cognitive and fatigue-related symptoms is essential for timely rehabilitative efforts.^36^ This is not only due to their association with HRQoL but also because of their impact on everyday functioning, including work capacity.^47^ Cognitive impairments and fatigue could however complicate patients’ ability to engage with and benefit from such interventions, underscoring the importance of tailoring rehabilitation strategies to individual cognitive and symptom profiles. We observed substantial symptom overlap between fatigue and symptoms of anxiety and depression, which further supports the need to address these conditions as part of the clinical management and rehabilitation. Importantly, fatigue, depression, and anxiety represent potentially treatable conditions. Interventions using blended cognitive behavioral therapy targeting fatigue in these patients have recently shown promising results also in this patient group.^48^ Our findings further highlight the need to systematically assess and rehabilitate cognitive symptoms across the disease trajectory. Cognitive rehabilitation is feasible in this population and can involve both restorative retraining and compensatory strategies.^49^ Such interventions should address overt deficits, such as memory and language, as well as more hidden but potentially disabling impairments, for instance, in executive functioning.

### Strengths and Limitations

The present analyses are based on cross-sectional data at each assessment time point, which limits inference about within-individual change over time, even though there was substantial overlap between cohorts across assessments. In particular, the long-term cohort encompassed a wide range of time since diagnosis, and in this cohort patients with oligodendrogliomas were overrepresented. In addition, the relationship between some cognitive domains and HRQoL could not be examined at all time points due to low numbers of cases with domain-specific impairments. The criteria used to classify cognitive impairment were relatively strict, meaning that more subtle cognitive difficulties that may still be clinically relevant for HRQoL were not captured in the analyses.^7^

A key strength of the present study is the inclusion of a relatively large and clinically representative cohort of patients with *IDH*-mutant gliomas, recruited with minimal selection bias. All patients underwent comprehensive, standardized neuropsychological assessments conducted by a neuropsychologist, allowing objective and domain-specific evaluation of cognitive functioning. This design enabled examination of specific cognitive domains, an approach that has rarely been applied in this population.

## Conclusion

This study supports a close relationship between cognitive functioning and HRQoL and demonstrates that this relationship is both domain-specific and time-dependent. The association is primarily driven by learning and memory at the time of diagnosis, while language and in particular executive functioning become more important determinants for global HRQoL over time. Fatigue is associated with both cognition and global HRQoL and may represent an important co-occurring factor. Importantly, targeting cognitive impairments, anxiety, depression, and fatigue may improve HRQoL in patients with *IDH*-mutant gliomas.

## Supplementary Material

npag049_Supplementary_Data

## Data Availability

Given the nature of this study, participants did not provide consent for public data sharing. Non-identified group-level data may be made available by the corresponding author upon reasonable request.
