# Supplementary material for: Contribution of cognitive function and fatigue to health-related quality of life in patients with IDH-mutant gliomas from diagnosis to long-term follow-up
Source: Neurooncol Pract. 2026 May 24;13(5):994–1005. doi: 10.1093/nop/npag049 (PMC13578484; doi:10.1093/nop/npag049)
Supplement: Supplementary file 1 [file npag049_supplementary_data.docx]

**Supplementary material**

**Supplementary table 1. Test descriptions**

| **Test** | **Description** |
| --- | --- |
| Boston Naming Test (BNT) | A confrontation naming task comprising 60 drawings of objects. The participant names each picture aloud, assessing lexical retrieval and language abilities. |
| D-KEFS Verbal Fluency; semantic fluency, and phonemic fluency | In the semantic condition, the participant generates as many words as possible within one minute from a semantic category (only animals). In the phonemic condition, words beginning with specific letters (F, A, S) are produced within one minute each. Both tasks assess verbal fluency and processing speed; the phonemic condition also involves executive control. |
| Rey Auditory Verbal  Learning Test (RAVLT) | A list of 15 words is read aloud five times, followed by immediate recall after each trial. After a 30-minute delay, a delayed recall trial is conducted. The test measures verbal learning, recall, and retention. |
| Rey Complex figure,  (RCFT) copying | Participants reproduce a complex geometric figure while viewing the original. The task assesses visuospatial constructional skills and planning aspects of executive functioning. |
| Brief Visuospatial Memory Test-Revised (BVMT-R) | Six geometric designs are displayed for 10 seconds on three consecutive trials. After each exposure, participants reproduce the figures from memory, followed by a delayed recall trial after 25 minutes. The task measures visuospatial learning and memory. |
| Trail Making Test, (TMT)  A and B | In part A, the participant connects numbered circles in ascending order, reflecting visual scanning, processing speed, and attention.  In part B, the participant alternates between numbers and letters (1–A–2–B…), assessing cognitive flexibility/executive functioning. |
| D-KEFS Color-Word Interference Test (CWIT) | All subtests should be performed as rapidly as possible.  CWIT 1. Naming colors, measures naming speed.  CWIT 3. Naming the color of incongruently colored words, measures verbal speed and response inhibition CWIT 4. Same as 3 but alternating between color naming and reading measuring aspects of cognitive flexibility. |
| WAIS-IV, Digit Span | A series of digits is read aloud with increasing length. In the forward condition, participants repeat the digits in the same order (short-term memory and attention). In the backward condition, digits are recalled in reverse order (working memory/executive functioning). |
| WAIS IV, Coding | Participants transcribe symbols paired with numbers using a provided key within a two-minute time limit. The task measures processing speed, sustained attention, and visuomotor coordination. |

| **Domain** | **Test variables** |
| --- | --- |
| Language | Boston Naming Test (BNT) 60 items, D-KEFS Verbal Fluency; semantic fluency, and phonemic fluency (only animals) |
| Learning/memory | Rey Auditory Verbal Learning Test (RAVLT) learning and delayed recall, Brief Visuospatial Memory Test-Revised (BVMT-R) learning and delayed recall. |
| Executive functioning | Trail Making Test (TMT) B, D-KEFS Color-Word Interference Test (CWIT) 3 and 4. |
| Attention/working memory | WAIS-IV, Digit Span forward and backward |
| Visuo-spatial functioning | Rey Complex figure (RCFT) copying |
| Processing speed | Trail Making Test, (TMT) A, WAIS-IV Coding |

**Supplementary table 2.
Neuropsychological test variables comprising the cognitive domains**

**Supplementary table 3. Hierarchical regression models for cognitive domains in relation to global HRQoL in patients with high fatigue preoperatively, one year postoperatively, and in the long-term cohort.**

|  |  | Global HRQoL  Pre-op  (n = 52) | Global HRQoL  One year  (n = 51) | Global HRQoL Long-term (n = 39) |
| --- | --- | --- | --- | --- |
|  |  | β | β | β |
| Block 1 | Sex  Age | 0.127 0.050  Adj. R² = -0.023 | 0.184  -0.168  Adj. R² = 0.014 | 0.170  -0.158  Adj. R² = -0.002 |
| Block 2 | Domain Language  Domain Learning/memory  Domain Executive  Domain Attention  Domain Visuospatial  Domain Speed | -0.279* -0.449** -0.010  0.183  0.070 NA  Adj. R² = 0.242  ΔR² = 0.328** | -0.005  0.272  -0.424*  0.076  -0.132  NA  Adj. R² = 0.082  ΔR² = 0.157 | 0.146  0.084  -0.422*  -0.257  0.092  -0.091  Adj. R² = 0.117  ΔR² = 0.252 |

*Note*. * = *p* <.05. **= *p* <.01 *** = *p* <.001. β = Standardized beta coefficient, Adj. R² = adjusted explained variance. ΔR² = change in unadjusted R² between blocks (F-change test). NA = not available because assumptions were not met.

**Supplementary table 4. Hierarchical regression models for fatigue domains in relation to global HRQoL preoperatively, 3 months postoperatively, one year postoperatively, and in the long-term cohort.**

|  |  | Global HRQoL  Pre-op  (n = 106) | Global HRQoL Three months (n = 107) | Global HRQoL  One year  (n = 106) | Global HRQoL Long-term (n = 82) |
| --- | --- | --- | --- | --- | --- |
|  |  | β | β | β | β |
| Block 1 | Sex  Age | 0.07 0.02  Adj. R² = -0.02 | 0.01 -0.17 Adj. R² = 0.01 | -0.02  -0.21  Adj. R² = 0.03 | 0.24  -0.31**  Adj. R² = 0.14 |
| Block 2 | General fatigue  Physical fatigue  Mental fatigue  Reduced activity  Reduced motivation | -0.14  -0.25*  -0.04  -0.21 -0.21*  Adj. R² = 0.48  ΔR² = 0.51*** | -0.35** -0.24* -0.19* 0.03 -0.25** Adj. R² =0.69 ΔR² = 0.68*** | -0.38**  -0.28* 0.00  0.01 -0.23*  Adj. R² = 0.61  ΔR² = 0.59*** | -0.51*** -0.02  -0.09  -0.07  -0.21  Adj. R² = 0.64  ΔR² = 0.51*** |

*Note*. * = *p* <.05. **= *p* <.01 *** = *p* <.001. β = Standardized beta coefficient, Adj. R² = adjusted explained variance. ΔR² = change in unadjusted R² between blocks (F-change test).

**Supplementary figure 1** Relation between fatigue, cognitive domains and *any cognitive impairment* preoperatively (A), at one year postoperatively (B), and in the long-term cohort (C).

**A.**

**B.**

**C.**

**Supplementary figure 2**
Heatmap of tumor location before first surgery in the preoperative cohort (n = 106).

**Supplementary figure 3**
Heatmap of tumor location before first surgery in the three-months cohort (n = 107).

**Supplementary figure 4**
Heatmap of tumor location before first surgery in the one-year group (n = 106).

**Supplementary figure 5**
Heatmap of tumor location before first surgery in the long-term cohort (n=78).
